# Hemolytic disease of the fetus and newborn and Rhesus alloimmunization in Latin American countries: a scoping review

**DOI:** 10.1186/s12884-024-07044-3

**Published:** 2024-12-20

**Authors:** Mário Dias Corrêa Júnior, Salvador Espino y Sosa, Milene Fernandes, Lais do Carmo, Renato Watanabe de Oliveira, Gabriela Kanevsky

**Affiliations:** 1 Department of Obstetrics and Gynecology, Minas Gerais Federal University, Campus Health, Av. Prof. Alfredo Balena, 190 - Santa Efigênia, Belo Horizonte, MG 30130-100 Brazil; 2 Clinical Research Department, Instituto Nacional de Perinatologia Isidro Espinosa de los Reyes, Mexico City, Mexico; 3 RWE and Late Phase, CTI Clinical Trial & Consulting Services, Lisbon, Portugal; 4 HEOR LATAM, Janssen-Cilag, São Paulo, Brazil; 5 RWE LATAM, Janssen-Cilag, São Paulo, Brazil; 6 Immunology LATAM, Janssen, Mendoza, Buenos Aires, CP (1428) 1259 Argentina

**Keywords:** Infant, Newborn, Anemia, Hemolytic, Rh alloimmunization, Erythroblastosis, Fetal, Latin America

## Abstract

**Background:**

Hemolytic disease of the fetus and newborn (HDFN) is a condition due to maternal blood group antibodies targeting antigens in fetal red blood cells, with significant prenatal/perinatal morbidity and mortality. Severe HDFN cases are often associated with alloimmunization against Rhesus D (RhD) or Kell antigens. Information about HDFN epidemiology and treatment in Latin American countries is limited. This review aims to identify and synthesize the available evidence on the epidemiology and management of HDFN in this region.

**Methods:**

In July 2023, EMBASE, PubMed, LILACS, and other databases were searched for articles reporting epidemiology, treatment, prenatal and perinatal outcomes, and patient journey of HDFN cases in Latin American countries. A snowball search of cross-references and gray literature complemented the initial search. Publications in English, Spanish, and Portuguese were reviewed. Data were extracted using a defined template and charted in tables.

**Results:**

We reviewed five guidelines and 19 observational studies from Brazil, Chile, Mexico, Argentina, Colombia, Panamá, Paraguay, and Peru. HDFN due to Rh alloimmunization ranged from 0.5 to 5 per 1000 live births, and anti-D remains the most frequent alloantibody type for severe HDFN. The perinatal mortality rate of HDFN is approximately 1.3–1.6 per 100,000 live births, and fetal deaths can reach 30% among patients treated with intrauterine transfusions. Up to 47% of alloimmunized pregnancies were referred to reference centers only during the third trimester. About 60% of eligible pregnancies received anti-D IgG prophylaxis.

**Conclusions:**

Although estimates in LATAM countries are scarce and lack standardized measures, we observed that the incidence, morbidity, and mortality of HDFN in this region are problematic. RhD alloimmunization was reported in approximately up to 70% of severe HDFN cases, despite anti D HDFN being largely preventable.

**Supplementary Information:**

The online version contains supplementary material available at 10.1186/s12884-024-07044-3.

## Background

Hemolytic disease of the fetus and newborn (HDFN), also called erythroblastosis fetalis, occurs in cases of red blood cell (RBC) incompatibility between pregnant individuals and their fetuses when a sufficient quantity of maternal alloantibodies crosses the placental barrier and binds to antigens at the surface of fetal RBCs with a strength that results in its destruction and subsequent anemia [1–3]. HDFN severity can range from mild (e.g. jaundice) to severe cases presenting with hydrops and fetal death or, after birth, kernicterus [2–4]. Pregnancies affected by severe HDFN often require repeated intrauterine transfusions (IUT), an invasive and risky procedure, and newborns need anemia monitoring and treatment for hyperbilirubinemia [3, 5].

Maternal alloimmunization often occurs in first pregnancies with RBC incompatibility when the maternal immune system encounters a fetal RBC antigen (for instance, during delivery) for the first time and produces antibodies. When these antibodies remain in the maternal serum, the individual is said to be alloimmunized. Although the ABO blood group system is the most frequent alloimmunization [6, 7], Rhesus (Rh) blood group is associated with the most severe cases of HDFN, particularly when resulting from D antigen [3, 8]. Other causes of HDFN include alloantibodies targeting other Rh antigens (such as c, C, E, and e), antigens of the Kell blood group (e.g., anti-K and anti-k), Kidd blood group (e.g., anti-Jka and anti-Jkb), Duffy blood group (e.g., anti-Fya), and MNS and s blood group. So far, HDFN has not been associated with antibodies against the P and Lewis blood groups [3, 8, 9].

The estimated worldwide prevalence of severe Rh disease in 2010 was 277/100,000 live births, contrasting with estimates of 2.5/100,000 live births in high-income countries with established perinatal/neonatal care [10]. Furthermore, the same study estimated a higher prevalence of Rh disease in low-to-middle income countries, namely, 525 in Eastern Europe/Central Asia, 386 in sub-Saharan Africa, and 345 in Latin America/Caribbean countries, per 100,000 live births [10, 11]. This gap between high-income and middle- to low-income countries can be explained by challenges in adequate IgG anti-D prophylaxis, and poor access to appropriate perinatal/neonatal care[5, 11, 12].

Few literature reviews have addressed the pathology of HDFN and the antenatal and postnatal treatment landscape, with most information being related to high-income countries [1, 3, 5]. Less is known about the epidemiology and treatment of HDFN in regions with a higher incidence, particularly in Latin America [11]. Understanding HDFN management and burden is relevant to promoting insight and debate about unmet needs. This is important to improve healthcare policies, patient care, and HDFN outcomes, as well as to assess opportunities for new therapies. Hence, we aimed to review and synthesize the available evidence and expert perspectives on the epidemiology, patient journey, and management of HDFN and Rh alloimmunization in this region.

## Methods

A scoping review framework was chosen to characterize the evidence related to the research question and to explore the range of estimates for selected epidemiological indicators [13]. The results are reported following the *Preferred Reporting Items for Systematic Reviews and Meta-Analyses Extension for Scoping Reviews* (PRISMA-ScR) recommendations [14].

### Identifying relevant studies

A literature search was conducted in April 2023 in the EMBASE, PubMed, and LILACS databases using free-text terms related to HDFN and Latin American descriptors [see Additional file 1]. A complementary search was performed in Value in Health Journal, Epistemonikos, and Google Scholar. The main inclusion criteria were full publications or gray literature (namely, theses/academic reports) reporting epidemiological or clinical studies (including case reports) of pregnant individuals and/or their fetuses, infants, or children affected by HDFN, and living in Latin American countries. In addition, a snowball method of cross-references was conducted until July 2023 to identify other relevant literature, including local publications in Portuguese or Spanish.

### Study selection

The main inclusion criteria were real-world evidence studies or registries on HDFN or Rh alloimmunization in LATAM reporting epidemiological estimates, clinical characteristics, or patient-reported outcomes published in the last 20 years in English, Portuguese, or Spanish. Clinical trials, clinical practice guidelines, literature reviews, and case studies were also reviewed for data extraction and citation searching, when applicable. The exclusion criteria were a study period before 2003 only, letters to the editor and commentaries, cohorts from non-LATAM countries, and preliminary gray literature if subsequent full peer-reviewed publications were available. Aggregated/ecological studies were included only if they presented national frequency estimates (the most recent were considered) or burden of disease measures (e.g., hospitalization characteristics). Two reviewers independently screened the titles/abstracts of the retrieved articles and then the full texts to identify eligible publications; discrepancies regarding the full texts were reviewed by a third reviewer.

### Data extraction

The data were extracted by two researchers (LC and MF) into a specific Excel form, namely study characteristics (author, year of publication, study period, country, study design), main eligibility criteria, and results regarding frequency of HDFN and maternal Rh alloimmunization, prenatal and neonatal treatment, mortality and clinical outcomes, burden of disease and awareness and patient journey. The extracted data were summarized qualitatively in Tables 1–3 and reviewed by all the authors.

**Table 1:** Summary of the included observational studies

Author, Year (Country)	Study period	Objectives	n	Patient population	n (%) with Rh alloimmunization	Type of Rh
Brítez, 2007 [15] (Paraguay)	2001–2004	To evaluate the prevalence of erythroblastosis fetalis caused by Rh incompatibility in the population of pregnant individuals who present at the hospital, patient costs, mortality rate and potential years of life lost	120	pregnant Rh-	28 (23%)	NR
Campos, 2016 [16] (Brazil)	2006–2014	To evaluate the correlation between anti-D antibody titers in pregnant women with a previous history of hemolytic disease and adverse pregnancy outcome	58	pregnant Rh alloimmunized	58 (all)	anti-D only (*n* = 23, 39.7%) anti-D + anti-C (*n* = 19, 32.8%) anti-D + other combinations (*n* = 16, 27.6%)
Choquepata, 2013 [17] (Peru)	2008–2012	To determine the clinical and laboratory characteristics of the newborns with hemolytic disease due to group incompatibility ABO blood or Rh factor and the prevalence of these	42	HDFN	7 (16.7%) [35 pts ABO incompatible]	NR
Duete, 2022 [18] (Brazil)	2017–2018	To assess the prevalence of maternal alloantibodies in pregnant women at a maternity hospital in northeastern Brazil and describe their perinatal outcomes	60	pregnant Rh alloimmunized	60 (all, 2.5%*)	> Of 45 medical records available from 60 pts: anti-D (*n* = 24, 53.3%), anti-C (*n* = 7, 15.5%), anti-c (*n* = 1, 2.2%), anti-E (*n* = 3, 6.7%), anti-Cw (*n* = 1, 2.2%), anti-K (*n* = 2, 4.4%), anti-Jka (*n* = 1, 2.2%), anti-M (*n* = 3, 6.7%), anti-Fya (*n* = 2, 4.4%), anti-Fyb (*n* = 1, 2.2%), Anti-Lea (*n* = 5, 11.1%), anti-Leb (*n* = 3, 6.7%), anti-Dia (*n* = 3, 6.7%)
Durango-Sanchez, 2023 [19] (Mexico)	2013–2018	To determine the maternal and fetal outcomes in pregnant women with Rh D incompatibility	250	pregnant Rh-	55 (22%)	all anti-D
Fernandes, 2021 [20] (Brazil)	2018–2020	To analyze the prevalence of maternal Rh alloimmunization from 2018 to 2020 in a maternity hospital in Amazonas	592	pregnant Rh-	592 (1.5%**)	NR
Lewis, 2018 [21] (Panamá)	2015–2016	To compare the impact of the implementation of interventions for the diagnosis of HDFN in a neonatology service	422	HDFN	14 (3.3%) [408 pts ABO incompatible]	NR
Lobato, 2008 [22] (Brazil)	1996–2006	To evaluate the relationship between obstetric history and RhD alloimmunization severity, employing the gestational age at the first intrauterine fetal transfusion as an indicator	82	HDFN with IUT	82 (all)	All anti-RhD antibody plus: anti-c (*n* = 6, 7.3%); anti-JKa/anti-JKb (*n* = 3, 3.6%); anti-Lewis (*n* = 2, 2.4%); anti-e (*n* = 2, 2.4%); anti-Kell (*n* = 1, 1.2%); anti-S (*n* = 1, 1.2%); anti-M (*n* = 1, 1.2%)
Lobo, 2007 [23] (Brazil)	2000–2005	To analyze the perinatal outcome of unrelated erythrocyte alloimmunization to the RhD antigen. To compare perinatal results in RhD alloimmunized vs. non-Rh-sensitized pregnant women	200	pregnant Rh-	55 (27.5%)	55/70 anti-D 15/70 other non-anti-D antibodies: Lewis (*n* = 7); Kell (3); MNS (3); Diego (2)
Nardozza, 2007 [24] (Brazil)	1995–2004	Evaluate and compare the perinatal mortality of Rh-negative pregnancies managed at São Paulo Federal University for 9 years, using either amniocentesis or MCA-PSV	291	pregnant Rh-	99 (34%)	all anti-D
Osanan, 2010 [25] (Brazil)	2007–2009	To determine prognostic factors of perinatal mortality in transfused fetuses	325	pregnant Rh alloimmunized	325 (all)	of patients with IUT: anti-D (*n* = 70, 54.5%), anti-D + anti-C (*n* = 42, 32.8%), anti-D + other combinations (*n* = 14, 10.9%), other non-anti-D (*n* = 2, 1.6%)
Pinochet, 2019 [26] (Chile)	2003–2019	To describe the results of the cases of fetal anemia that required intrauterine transfusion at the reference center, between 2003 and 2019	17	severe HDFN with IUT	11 (65%) [pts ABO incompatible NR]	NR
Ramírez-Robles, 2010 [27] (Mexico)	1987–2008	To review the perinatal outcome with intrauterine transfusion (IUT) in severe alloimmunization RhD over 21 years in a referral center in México	150	HDFN with IUT	150 (all)	all anti-D
Roldán-Isaza, 2023 [28] (Colombia)	2014–2018	To describe the clinical and epidemiological profile of patients with HDFN treated in a university hospital during the 2014–2018 period	216	HDFN	NR [pts ABO incompatible NR]	NR
Sá, 2009 [29] (Brazil)	1997–2007	To determine the incidence of AE associated with EXT performed during the past ten years and to evaluate if there is an association between the severity of patient’s clinical condition before the procedure and the incidence of AE	300	HDFN due to RhD with EXT	300 (all)	all anti-D
Seidl, 2013 [30] (Brazil)	2006–2009	To identify the major risk factors related to EXT in pregnancies afflicted with HDFN and to evaluate the effect of applied therapy	124	HDFN due to RhD	124 (all)	all anti-D
Sepúlveda-Martínez, 2013 [31] (Chile)	2004–2009	To compare neonatal and six months of life morbidity of babies affected by Rh alloimmunization during pregnancy that required at least one intrauterine blood transfusion, with babies that did not require that procedure	857	pregnant Rh-	23 (2.7%**)	all anti-D
Torres, 2011 [32] (Argentina)	2006–2010	To evaluate the effectiveness of IVIgG treatment for severe HDFN	94	severe HDFN with IUT	94 (all)	NR
Villaschi, 2012 [33] (Brazil)	2005–2011	To evaluate birth conditions, treatment, and outcome of newborns with HDFN by Rh alloimmunization after delivery, and during hospital stay. To compare the postnatal clinical course of those who underwent transfusion procedures during pregnancy with those who did not receive IUT	114	pregnant Rh alloimmunized	114 (all) Note: 75 fetuses HDFN	anti-D (*n* = 38, 55.9%), anti D + C (*n* = 20, 29.4%), anti D + C + other (*n* = 4, 5.9%), anti D + C + Kell + other (*n* = 2, 2.9%), anti D + Kell + other (*n* = 1, 1.5%), anti D + other (*n* = 2, 2.9%); other (*n* = 1, 1.5%)

*Abbreviations*: *AE* adverse events, *EXT* exchange transfusion, *HDFN* hemolytic disease of the fetus and newborn, *IUT* intrauterine transfusion, *IVIgG* intravenous immunoglobulin G, *MCA-PSV* middle cerebral artery peak systolic velocity, *n* number, *NR* not reported, *pts* patients, *Rh* Rhesus

^a^Overall prevalence in all antibody screens performed from Jan 2017 to Oct 2018 (*n* = 2391). ^b^Frequency among pregnant women

## Results

### Literature overview

**Fig. 1:**
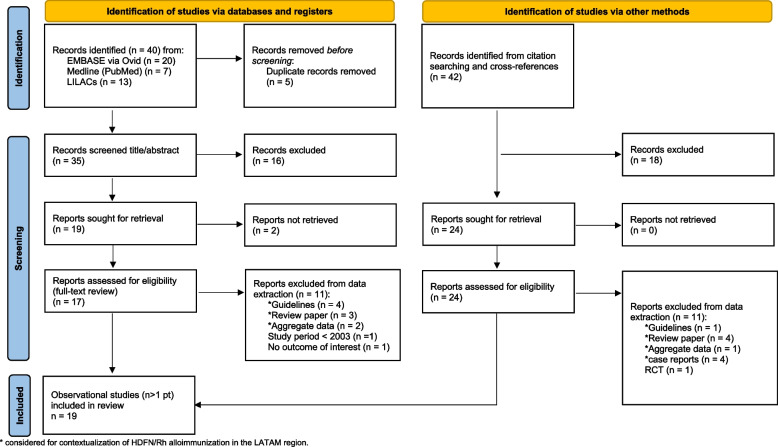
PRISMA flowchart of the literature review

Following deduplication, 35 records were screened, and 17 potentially met the eligibility criteria after review of the title/abstract (Fig. 1). From 42 other records identified from cross-references, 24 were selected for full-text review.

The available evidence on HDFN in Latin America is scarce, heterogeneous, and based on retrospective studies (*n* = 13), thesis/academic reports (*n* = 6), and case reports (*n* = 2). The observational studies (*n* = 19, Table 1), including retrospective studies and thesis, were from Brazil (*n* = 10), Chile (*n* = 2), Mexico (*n* = 2), Argentina, Colombia, Panamá, Paraguay, and Peru (one study per country). One study reported funding from non-governmental organizations other than the pharmaceutical industry [29].

Among the retrospective studies that included only HDFN patients (*n* = 7), three evaluated HDFN in general (including ABO alloimmunization cases) [17, 21, 34], two evaluated severe HDFN [26, 35], and another two evaluated HDFN due to Rh alloimmunization [29, 30]. Of retrospective studies with pregnant individuals (*n* = 12), five included Rh-negative pregnant individuals with obstetric history of RhD alloimmunization [16, 20, 22, 25, 33], four studies included all Rh-negative pregnant individuals with Rh incompatibility attending reference centers [15, 19, 23, 24], two studies included pregnancies with severe RhD alloimmunization receiving IUT [27, 31], and one identified positive alloantibody screens [18].

In addition to observational studies, five clinical guidelines related to HDFN management were also identified, and two aggregate analyses from Brazil were considered for data extraction related to disease burden/costs [36, 37].

### Frequency of HDFN, in general, and due to Rh alloimmunization

Epidemiological estimates of HDFN in LATAM countries are scarce and lack standardization. A study from Peru identified 42 newborns with HDFN due to ABO or Rh alloimmunization between 2008 and 2012, corresponding to an incidence of 3.4:1000 live births [17]. In that study, Rh alloimmunization was reported in 16.7% of newborns with HDFN, and the authors estimate HDFN rates due to ABO and Rh alloimmunization of 2.9 and 0.5 per 1000 newborns, respectively. In Panama, of 422 newborns with HDFN during 2015 and 2016, 96.7% were due to ABO alloimmunization and 3.3% due to Rh alloimmunization [21]. A study from Chile with 17 cases of fetal anemia with IUT (i.e., severe HDFN) indicated that 11 cases were due to Rh alloimmunization (64.7%) [26]. In a reference center from Paraguay, Rh alloimmunization was reported in 28 (22%) newborns out of 128 pregnancies with Rh incompatibility followed from January 2002 to December 2004 [15].

The available data may be underestimated due to diagnostic failure, underreporting of cases, and the lack of a systematic investigation and monitoring policy for these conditions [22].

### Frequency of maternal Rh alloimmunization

Overall, the frequency of Rh alloimmunization ranged between 1.5% and 3.8% of pregnancies [18, 20, 31] (Table 1). Considering the studies with identified Rh-negative women, the proportions of Rh alloimmunized pregnant women followed in reference centers were 22% in Mexico and 34% in Brazil [19, 23, 24] (Table 1).

Among 45 patients with a positive screen of alloantibodies between January 2017 and October 2018 in one center from Brazil, anti-D was the most frequent antibody (53.3%), followed by anti-C (15.5%), anti-E (6.7%), anti-M (6.7%), anti-Leb (6.7%), and anti-Dia (6.7%), in addition to other less frequent antibodies [18]. In another report from Brazil between 2006 and 2014, considering 58 high-risk pregnancies of individuals with prior obstetric history of moderate or severe HDFN caused by anti-D alloantibodies, 39.7% were due to anti-D only, 32.8% presented anti-D and anti-C, and 27.6% had a combination of other antibodies [16]. Of pregnancies receiving IUT at a third Brazilian center, approximately 55% had anti-D antibodies only, 33% had anti-D and anti-C, 11% had anti-D + other combinations, and 2% had other non-anti-D antibodies [25, 33].

### Prenatal and neonatal treatment

#### Intrauterine transfusions

The IUT alternatives reported were intraperitoneal, hepatic vascular, and cord vascular transfusions. The number of Rh alloimmunized pregnancies receiving IUT was reported in 11 studies, and three included only patients with IUT (Table 2). The highest proportion of cases receiving IUT was reported in a single-center study with severe HDFN population in Argentina, where 87% of patients received IUT [32]. In studies with IUT and available information (*n* = 8), the mean number of transfusions per patient ranged between 1.6 and 3.5, and an average of 4 transfusions per fetus (range 1 to 9) were reported for fetuses with identified hydrops [27]. In five studies reporting IUT-related deaths, the mortality rate varied between 1.9% and 3.7% of transfusions.

**Table 2:** Prenatal and neonatal treatment and complications

Author, Year (Country)	n	patient population	n Rh allo pts	% with IgG anti-D	% Rh allo with IUT	n of IUT/ mean (range)	Weeks at first IUT	IUT deaths	IUT complications	prenatal complications	premature births	newborn complications	newborn treatment
Brítez, 2007 [15] (Paraguay)	120	pregnant Rh-	28	NR	NR					NR	< 24w: 25%	deaths: 17% anemia: 51.7% hospital stay: 8.8 days	EXT: 24.1% Phototherapy: 4.1 days
Campos, 2016 [16] (Brazil)	58	pregnant Rh alloimmunized	58	NR	48%	97/3.5 (1–8)	25.1	2.1%	premature birth of patients with IUT < 37w: 100% < 29w: 4.5%	Anemia: 12.1% Fetal deaths: 12.1%	< 37w: 62.8% < 29w: 2.0%	no neonatal deaths neonatal stay: 15.7 days	EXT: 66.7% Phototherapy: 94.1%
Choquepata, 2013 [17] (Peru)	42	HDFN	7	NR	NR					NR	< 37w: 16.7% - all ABO incompatibility	anemia: 71.4% (Rh); 51.4% (ABO) hepatomegaly: 42.9%(Rh), 45.7% (ABO) sepsis: 66.7% (Rh); 42.9% (ABO)	NR for Rh
Duete, 2022 [18] (Brazil)	84	pregnant Rh alloimmunized	60 (41 other than only anti-D)	–	5%	NR/NR	NR	NR	NR	Of 41 non-anti-D cases: anemia: 4, Fetal death: 2.4%	NR	Any intervention: 83.3% of 18 with positive DAT	EXT: 2/41 Phototherapy: 14/41
Durango-Sanchez, 2023 [19] (Mexico)	250	pregnant Rh-	55	73.6% of eligible patients	4%	NR/NR	NR	NR	NR	NR	median: 38w (range: 29–41)	deaths: 1.2% reanimation: 2% neonatal UCI: 3.1%	EXT: 11.8% Phototherapy: 47%
Fernandes, 2021 [20] (Brazil)	592	pregnant Rh-	592	58.8%	NR					Fetal death 0.73%	NR	NR	NR
Lewis, 2018 [21] (Panamá)	422	HDFN	14	NR						NR	< 37w: 0.7%	NR	EXT: 4.7% (*n* = 20)
Lobato, 2008 [22] (Brazil)	82	HDFN with IUT	82	–	100%	NR/NR	< 24w: 15.8%	NR	NR	Fetal death: 2.4% ^a)^	NR	Neonatal deaths: 4 (4.9%)	NR
Lobo, 2007 [23] (Brazil)	200	pregnant Rh-	70	NR	17%	NR/NR	NR	NR	NR	Ultrasound changes: 24.3%	< 37w: 60%	Neonatal deaths: 6 (8.6%) > 3 days hospitalization: 43 (61.4%)	IUT and/or EXT: 60.0%
Nardozza, 2007 [24] (Brazil)	291	pregnant Rh-	99	NR	25%	NR/NR	NR	NR	NR	Fetal death: 7% Hydrops: *n* = 13 Pleural effusion: 1 Cardiac effusion: 1 Ascites: 13	Mean: 35.9w	Neonatal deaths: 5%	NR
Osanan, 2010 [25] (Brazil)	325	pregnant Rh alloimmunized	325	–	39%	305/2.4 (NR)	28.0	2.0%	Bradycardia: 9.5% Umbilical cord Thrombosis (*n* = 9) Amniorrhexis (3) Chorioamnionitis (3) Premature birth (4)	Fetal death: 13%	NR	Hydrops: 34.4% Deaths: 7/120 (5.8%)	NR
Pinochet, 2019 [26] (Chile)	17	severe HDFN with IUT	11	NR	100%	27 in 17 pts/1.6 (1–5)	28	3.7%	Premature birth: 7.4% Chorioamnionitis: 3.7%	–	mean: 32w	NR	EXT 24 h life: 80% repeat EXT during ICU stay: 60%
Ramírez-Robles, 2010 [27] (Mexico)	150	HDFN with IUT	150	–	100%	531/3.5 (1–9)	> 28w: 22.7%	1.9%	NR	Hydrops: 45% Fetal death: 18%	median: 36w	Neonatal death: 10 (7%)	EXT: 23% other transfusions: 18%
Roldán-Isaza, 2023 [28] (Colombia)	216	HDFN	–	65.6%	NR					NR	mean: 38.1w	Icteric: 85.1% Reanimation: 16.7% ARDS: 7.9% hospital stay: 98.1% [8.2d] ICU stay: 19.9% [4.9d]	EXT: 10.3% Phototherapy: 86.4%
Sá, 2009 [29] (Brazil)	300	HDFN due to RhD	300	–	23%	NR/NR	NR	NR	NR	NR	mean: 35.7w	NR	EXT: 47.7% [total EXT: 207, 38% pts with AE, 1 death]
Seidl, 2013 [30] (Brazil)	124	HDFN due to Rh-D	124	–	25%	NR/NR	NR	NR	NR	Hydrops: *n* = 6	mean: 35.9w	NR	EXT: 50%
Sepúlveda-Martínez, 2013 [31] (Chile)	857	pregnant Rh-	23	NR	39%	NR/2 (1–7)	NR	NR	needed EXT: 30%, gestational age at birth: 34.4w	Hydrops: *n* = 1	mean: 36w	Neonatal deaths: 2 (8.7%)	EXT: 20% Phototherapy: 76%
Torres, 2011 [32] (Argentina)	94	HDFN	94	–	87%	184/2.2 (NR)	NR	0%	no complications	Hydrops: *n* = 34	mean: 34w	Perinatal deaths: 14.6%	NR
Villaschi, 2012 [33] (Brazil)	114	pregnant Rh- alloimmunized	114 (75 fetus HDFN)	–	41.3%	81/2.6 (1–5)	NR	NR	NR	Hydrops: *n* = 12	median: 36w < 37w: 58.1%	Hydrops: 10.7% Reanimation: 54.7% ARDS: 45.3% sepsis: 21.3% death: 5.3% hospital stay: 77.3%	EXT: 45.3% [total EXT, 56] Phototherapy: 95% no treatment: 4%

*Abbreviations*: *AE* adverse events, *RDS* acute respiratory distress syndrome, *DAT* direct antiglobulin test, *EXT* exchange transfusion, *HDFN* hemolytic disease of the fetus and newborn, *ICU* intensive care unit, *IUT* intrauterine transfusion, *IgG* immunoglobulin G, *n* number, *NR* not reported, *pts* patients, *Rh* Rhesus, *Rh* *allo *Rh-alloimmunized, *w* week

^a^Excluded pregnancies with hydrops fetalis or other fetal abnormalities

Following IUT, premature births (gestational age < 37 weeks) were induced in 20% to 100% of patients [16, 26], approximately 35% of cases remained hydropic [33] and 30% to 50% of neonates still required exchange transfusion after birth despite having received IUTs [30, 31]. Ramírez-Robles described the perinatal outcomes associated with IUTs in severe RhD alloimmunization over 21 years (1987–2007) in a referral center in México [27]. During this period, 150 fetuses received a total of 531 IUT. Of the 27 fetal deaths, 10 (37%) were due to IUT complications despite the low IUT-related mortality rate (2% per procedure). Two centers in Brazil reported identical IUT-related mortality rates of 2% per procedure [16, 25]. One case report from Costa Rica reported that despite fetal hydrops developing at an early stage of pregnancy, the perinatal outcome was satisfactory following 6 IUT although delivery was induced at 36 weeks [38]. Lobato et al. reported data about 82 RhD alloimmunized pregnancies that received an IUT between July 1996 and June 2006, 92.7% resulted in live newborns, 2.4% were stillborn, and 4.9% were neonatal deaths [22]. Regarding the risk factors for HDFN, 76.8% of pregnancies had at least one previous sensitizing event, namely, more than one previous pregnancy [median (percentiles 5 e 95): 3 (1–6)], stillbirth (34.2%), neonatal death (40.2%), fetal or neonatal hydrops (17.1%), or IUT (13.4%), and 44.8% had a previous history of neonatal exchange transfusion (44.8%) [22]. These risk factors were associated with earlier gestational age at the first IUT. Of note, the authors excluded severe cases with hydrops fetalis or other fetal abnormalities.

#### Neonatal treatment

A total of 13 studies reported treatment of neonates born to Rh-negative alloimmunized women, of which 6 studies have included specifically neonates with HDFN due to Rh alloimmunization (Table 2).

In Fortaleza, Brazil, 83.3% of newborns with positive direct antiglobulin test results required an intervention, namely, phototherapy (*n* = 14), blood transfusion (*n* = 2), or exchange transfusion (*n* = 2) [18]. Other studies in São Paulo, Brazil, revealed that 60% to 67% of newborns from Rh alloimmunized women had IUT or exchange transfusions [16, 27]. In Paraguay, of 28 newborns with a positive direct antiglobulin test, 7 (25%) received exchange transfusions [15]. Another study in Chile indicated that 13% of newborns affected by Rh alloimunization required exchange transfusion, corresponding to 30% of the 7 fetuses with IUT [31].

### Mortality and Clinical Outcomes

Fetal deaths were reported in nine studies, ranging from 0.73% (in Rh alloimmunized pregnancies in Manaus, Brazil) to 31% (in patients with IUT). On the other hand, neonatal or perinatal deaths were reported in 11 studies, from no observed deaths up to 17% [15]. The presence of hydrops significantly increases fetal mortality (30% vs 8%, RR: 3.5 [95% CI 1.6 to 7.9]) [27].

In Brazil, among pregnancies complicated by Rh alloimmunization, a fetal mortality rate of 10.5% was reported during the period 2005–2011 [33]. The same study reported that 10.7% of live births were hydropic and that major neonatal complications included resuscitation in the delivery room (54.7%), respiratory distress syndrome (45.3%), sepsis (21.3%), and death (5.3%). Hospitalization at the neonatal intensive care unit (ICU) was necessary for 77.3% of live births [33]. In Paraguay, of 28 newborns of pregnancies with Rh alloimmunization, 15 (52%) had anemia and 5 (17%) died [15].

Some studies have also evaluated other blood group incompatibilities. Of 42 newborns with HDFN identified in one regional hospital in Peru, from 2008–2012, 16.7% were born from mothers with Rh incompatibility and 83.3% from mothers with ABO incompatibility [17]. However, more HDFN newborns from mothers with Rh incompatibility had anemia (71% vs. 51% from mothers with ABO incompatibility), sepsis (67% vs. 43%), and pneumonia (33% vs. 7%). The authors of this study suggested that the proportion of HDFN cases due to Rh alloimmunization was smaller after the implementation of Rh immunoprophylaxis. In Brazil, of 41 cases with non-anti-D alloimmunization, 4 with fetal anemia were observed, and one fetal death occurred in a patient whose mother had anti-M antibodies [18]. Although anti-M antibodies are less frequently associated with clinically relevant HDFN, one case report from Colombia highlighted the importance of testing against other blood group antigens [39].

### Burden of Disease and Costs

Between 2014 and 2019, a total of 17,185 hospitalizations due to HDFN were registered in the database of the Information Technology Department of the Brazil Public Healthcare (DATASUS), with a mean duration of 5.3 days; 64 fetal deaths were registered [36]. In her thesis, Sá et al. estimated that HDFN due to RhD alloimmunization would account for 3.2 disability-adjusted life years (DALY) in 2007–2010. Although lower than other perinatal diseases (e.g., 120 DALYs for asphyxia and trauma), higher values were estimated in the poorest regions of Brazil (North and Northeast) [37]. Of note, 2,803 hospitalizations for HDFN were registered in Brazil in 2022, in DATASUS [40].

In 2007, in Paraguay, Britez estimated that the costs (direct and indirect, by pregnancy and assuming 3-day hospitalizations for pregnant individuals and 9-day hospitalizations of newborns) associated with HDFN would reach up to US $879, or even US $105,148 when adding the years of productive life potentially lost [15].

### Guidelines, Referral, and Diagnosis

Most guidelines related to anti-D immunoprophylaxis and HDFN treatment were developed or updated in the last 5 years, except in Argentina (published in 2010) and Chile (2015) – Table 3. The guidelines from Uruguay were independent of the country's health ministry/agency.

**Table 3:** Summary of relevant maternal guidelines in LATAM countries

Title	Country	Year	IgG anti-D recommendation	IUT recommendation
*Guía de manejo obstétrico y del recién nacido en madre aloinmunizada*	Uruguay	2021	• Within 72 h of a potentially immunizing event: 1st trimester: 120 µg IV or IM 2nd and 3rd trimester: 120 µg IV or 300 µg IM to all women RhD negative from 28 to 32w, according to ministerial recommendation • Postpartum: one dose of 120 µg IV or 300 µg IM, in the first 72 h	If MCA-PSV > 1.5 MoM and < 35w and 6 days: “repeat within 24 to 48 h hours, to reduce false positive (…) and depending on gestational age, perform a diagnostic cordocentesis and eventually therapy or termination of pregnancy.”
*Enfermedad Hemolítica Perinatal Control Inmunohematológico y Profilaxis*	Argentina	2010	• All RhD-negative women are not sensitized 28-32w: one dose ≥ 250 µg IM or IV • If sensitizing event: 1st trimester: one dose ≥ 100 µg IM or IV 2nd and 3rd trimester: one dose ≥ 250 µg IM or IV • Postpartum until 72 h Dose calculated according to the magnitude of bleeding: one dose ≥ 250 µg IV or IM Without quantification of fetomaternal hemorrhage: one dose ≥ 300 µg IV or IM	NR
*Diagnóstico y Tratamiento de la Enfermedad Hemolítica por Isoinmunización a Rh en el Recién Nacido*	Mexico	2018	Nonsensitized Rh-negative woman who intends to become pregnant or who is pregnant, except if the father of the newborn is also Rh negative Dosage: 100 μg (500 IU), in the first pregnancy	NR
*Manual de Gestação de Alto Risco*	Brazil	2022	Nonsensitized Rh-negative women: 300 μg at the 28w, up to 72 h postpartum of an Rh + or unknown Rh factor, and up to 72 h after a procedure/event that leads to risk of maternal sensitization	If fetal anemia based on MCA-PSV > 34w: delivery < 34 weeks: IUT if presenting hydrops fetalis or isolated ascites
*Guía Perinatal*	Chile	2015	• Nonsensitized Rh-negative women, within 72 h postpartum, upon confirmation of Rh ( +) • For pregnancies longer than 40w, a second dose is recommended after 40w if the previous dose was more than 12 weeks ago • Woman at risk of fetomaternal hemorrhage • Prophylaxis is not recommended for women with spontaneous abortion < 12w Dose: 300 μg IM (adjusted if fetomaternal hemorrhage)	IUT can be considered if MCA-PSV > 1.5MoM and Fetal Hematocrit ≤ 30%

*Abbreviations*: *IgG* immunoglobulin G, *IM* intramuscular, *IU* international units, *IUT* intrauterine transfusion, *IV* intravenous, *MCA-PSV* middle cerebral artery peak systolic velocity, *MoM* multiples of the median, *NR* not reported, *Rh* Rhesus, *w* week

All guidelines referred to IgG anti-D immunoprophylaxis. Guidelines from Uruguay, Argentina, Brazil and Chile referred to administration within 72 h of postpartum, and after a potentially immunizing event (except Chile guidelines). Mexico guidelines recommended the prenatal administration of anti-D immunoprophylaxis to nonsensitized Rh-negative woman who intends to become pregnant or who is pregnant (except if the father of the newborn is also Rh negative), despite also reporting evidence of combined prenatal and postnatal prophylaxis.

Three guidelines mentioned criteria for IUT decision. Rh-negative alloimmunized patients should be managed through Doppler evaluation of the middle cerebral artery peak systolic velocity (MCA-PSV), and repeated weekly when they are at high risk of fetal anemia [16, 24, 25]. Cordocentesis should be performed between 20 and 34 weeks in cases with hydrops fetalis or changes in MCA-PSV, and IUT is indicated for up to 34 weeks when the fetal hemoglobin deficit is > 5 g/dL [16, 24, 25]. Only the Mexican guidelines presented recommendations regarding neonatal treatment, including phototherapy and exchange transfusions. Phototherapy is recommended in newborns with hyperbilirubinemia, based on gestational age and bilirubin levels. Exchange transfusions are recommended in the presence of acute encephalopathy and lack of bilirubin control.

Other studies evaluated awareness and patient journey. In a study from Brazil, most (89.5%) health professionals indicated that the indirect antiglobulin test was the best test for identifying alloimmunization during pregnancy and postpartum [35]. In addition, the mean knowledge score about childbirth in pregnant women at risk of becoming alloimmunized to RhD was less than 80%, corresponding to inadequate knowledge [35]. A delay in the referral of alloimmunized pregnant women to high-quality specialized prenatal services (preferably fetal medicine) was reported in other Brazilian studies, with up to 47% of the pregnant women referred only after the third trimester [33, 41]. In addition, one study from Brazil-Amazonas indicated a mean number of prenatal consultations for alloimmunized pregnancies of 5.3, below the recommendations of WHO and the Brazilian Ministry of Health (minimum of 6 consultations for pregnancies in general) [20].

#### IgG anti-D prophylaxis

Three studies with Rh-negative pregnant individuals eligible for IgG anti-D reported frequencies of prophylaxis: 59% of 265 pregnancies in Brazil-Amazonas [20], 66% of 61 pregnancies in Colombia [34], and 74% of 163 pregnancies in Mexico [19]. Notably, a lack of access was the reason for 3.7% of the Rh-negative pregnant women not receiving IgG anti-D [20].

## Discussion

HDFN continues to impose a significant burden worldwide and in LATAM countries. We observed that the incidence of HDFN in LATAM due to Rh alloimmunization ranged from 0.5 to 5 per 1,000 live births, and anti-D remains the most frequent alloimmunization in severe HDFN. In the USA, the prevalence of any HDFN was estimated to be 1,695 per 100,000 live births, with ABO and Rh alloimmunization accounting for 78.1% and 4.3% of cases, respectively, in addition to other antigens (17.6%) [42]. The Rh-induced HDFN live birth prevalence rates decreased from 0.99 per 1,000 births during 1996–2000 to 0.44 per 1,000 births in 2006–2010 [42]. In Brazil, Nardozza et al. considered that the significant incidence of HDFN is probably due to the lack of awareness and the high cost of prophylaxis [24]. In fact, although the antenatal and postnatal anti-D prophylaxis was defined in several guidelines and center protocols in the LATAM region, we observed adherence rates up to 74%, while another review identified adherence rates of 80% to 90% for antenatal prophylaxis and 95% to 100% for postnatal prophylaxis, in high-income countries (UK, Australia, Canada, and the US) [43].

HDFN caused by Rh alloimmunization is more likely to require medical interventions, including simple or exchange transfusions, and to result in preterm delivery [42]. We observed that pregnancies complicated by Rh alloimmunization in the LATAM region present a considerable fetal mortality rate, especially in the presence of hydrops, and that most births are premature and /or delivered by cesarean section, which is also a recommendation from local guidelines. Currently, the only recommended treatment is IUT, which is an extremely complex and invasive procedure performed at reference centers since the procedure-related mortality rate could reach 4% or even higher if repeated procedures are needed. A recent meta-analysis revealed that the mean gestational age at first IUT was between 25 and 27 weeks and that 14.8% of pregnancies with HDFN due to RhD treated with IUT presented hydrops fetalis, with a mean fetal mortality rate of 19.8% ± 29.4% and a mean gestational age at birth between 34 and 36 weeks [3]. We observed that, in pregnancies receiving IUT in the LATAM region, fetal death occurred still in up to 18% of cases, and that up to half of the newborns from these pregnancies needed complimentary post-partum exchange transfusions. A recently published study from a single center in Brazil identified 36 deaths (14 intrauterine and 22 neonatal) among 169 fetuses of alloimmunized pregnant women who received 388 IUTs between January 1991 and June 2021 [44]. Other IUT complications were also reported, namely post-transfusion cord bleeding, fetal bradycardia, premature rupture of ovular membranes, and emergency cesarean section [44]. New treatment options with proven efficacy and less invasive administration are needed for severe HDFN patients.

Neonatal or perinatal deaths occurred in up to 17% of pregnancies with alloimmunization, and resuscitation procedures were deemed necessary in up to 55% of cases. One systematic review, with most studies from middle- to high-income countries, observed an overall neonatal mortality rate of up to 6%, higher among infants treated with exchange transfusions, and who received IUT [5]. Furthermore, newborns with HDFN usually require longer hospitalizations and additional procedures in a highly specialized healthcare setting. In addition to phototherapy, exchange transfusions are often needed and performed in LATAM, but this procedure also raises safety concerns, with estimates that one-third of patients will have at least one procedure-related adverse event. Furthermore, a retrospective cohort study in the Netherlands also highlighted that the incidence of sepsis among neonates with HDFN remains high, especially among those with a central-line required for post-partum exchange transfusions [45].

We observed proportions between 22 and 34% of Rh alloimmunization among Rh-negative pregnant women. These higher proportions than those reported in other world regions can be explained by the fact that included studies were from reference centers in LATAM following more severe cases, but also by a lower uptake of Rh immunoprophylaxis in LATAM countries. A recent study estimated that post-partum immunoprophylaxis was fully adequate in 2 LATAM countries (i.e. 6% of that geographical region) while 22 countries (71% of LATAM region) had an uptake of post-partum immunoprophylaxis lower than 80% [12]. In other world regions, routine postnatal administration of Rh immunoglobulin has significantly reduced the risk of maternal alloimmunization to about 1 to 3 in 1000 RhD-negative pregnant individuals [1]. For instance, before routine antenatal anti-D prophylaxis, this was the third most common alloantibody in pregnancies in Iceland (12.5% of detected alloantibodies) and the incidence of RhD alloimmunization was 6.1 per 1000 births of RhD negative women [46, 47]. To avoid adverse perinatal outcomes, it is critical to reinforce the use of immunoprophylaxis for anti-D alloimmunization in pregnant individuals. Additionally, given the association of non-anti-D antibodies with the development of HDFN, it may be warranted to request an antibody screen (indirect antiglobulin test) for all pregnant individuals, even those who are D-positive, and direct antiglobulin test for newborns whose mothers have an alloantibody other than anti-D, to diagnose non-anti-D maternal alloimmunization [18]. Better education for pregnant individuals who are Rh-negative during antenatal care may enhance adherence to prophylaxis and inform family planning [24, 35, 48].

Developing local clinical guidelines based on evidence and best practices to improve patient care is important. This could promote better knowledge of anti-D prophylaxis, help recognize risk factors in obstetric history, and properly analyze and request additional tests [49]. Awareness of primary care health professionals is key to early referral to reference centers and is important to avoid delayed diagnosis of fetal conditions and initiation of IUT to control anemia [33, 41, 50]. For instance, after the introduction of routine first-trimester antibody screening in the Netherlands, in 1998, the incidence of hydrops declined significantly from 39 to 15%, and the incidence of severe hydrops decreased from 18 to 4% [51]. Hence, local policies and guidelines should clearly define strategies for identification and referral of Rh alloimmunization in pregnancies, adequate antenatal care, and post-natal management, to mitigate challenges with healthcare access and missed opportunities for HDFN prevention. There is much room for improvement in all LATAM countries, namely by updating guidelines and incorporating the best evidence and international recommendations [52].

### Strengths and limitations

A strength of this review is the minimal limitation of the study design criteria and the extensive literature search by including a snowball method. However, as most studies were single-center retrospective chart reviews and few studies collected data from the last decade, our findings may not be representative of the full population in the LATAM region. On the other hand, all studies were from reference centers, which may lead to an underestimation of the true incidence of HDFN and Rh alloimmunization due to a lack of information on pregnancies followed by general clinicians, midwives, or other healthcare practitioners. We decided to include theses for a more comprehensive understanding of HDFN in LATAM, as they describe original research that has not been published in peer-reviewed journals, sometimes from countries with no other information (such as Peru). However, we recognize that this type of gray literature may undergo less rigorous scrutiny compared to published peer-reviewed articles and, since they may not be easily accessible, some theses and dissertations may have been missed despite the extensive snowball search performed.

We observed high heterogeneity among the study populations. For instance, the HDFN population overall vs. the HDFN population due to RhD alloimmunization vs. maternal alloimmunization overall vs. case reports of non-anti-D alloimmunization. This heterogeneity was also found in available treatment options, the prevalence of RhD alloimmunization, and sociodemographic differences that may affect the reported incidences of, for example, IUT, fetal deaths, and hydrops. Finally, in our review, most studies on HDFN in Latin America were earlier than 2015 and we cannot exclude that more recent studies may have been published since the review cut-off date in 2023.

Future multinational studies are required to determine the incidence of HDFN due to Rh alloimmunization and associated risk factors in populations with diverse sociodemographic and genetic characteristics. The treatment pattern, access to healthcare resources, and clinical burden of HDFN, in terms of perinatal and long-term outcomes, should also be further evaluated.

## Conclusions

The incidence, morbidity, and mortality of HDFN in the LATAM region are troublesome, and RhD alloimmunization is reported in up to approximately 70% of severe HDFN cases. Rh immunoglobulin immunoprophylaxis to prevent sensitization to the D antigen is the most impactful intervention to reduce severe cases of HDFN. For women with Rh alloimmunization or with alloimmunization to other antigens associated with poor outcomes, early identification of pregnancies at risk of severe HDFN, efficient prevention programs, and timely referral to reference centers can significantly reduce the burden of this condition. The findings from this review are a contribute to raise awareness and strengthen public healthcare policies on HDFN.

## Supplementary Information

Supplementary Material 1: Search strategies used in the databases.

Supplementary Material 2.

## Data Availability

All the data generated or analyzed during this study are included in this published article.

## Abbreviations

AE: Adverse events
ARDS: Acute respiratory distress syndrome
EXT: Exchange transfusion
HDFN: Hemolytic disease of the fetus and newborn
ICU: Intensive care unit
Ig: Immunoglobulin
IM: Intramuscular
IUT: Intrauterine transfusions
IV: Intravenous
LATAM: Latin America
MCA-PSV: Middle cerebral artery peak systolic velocity
MoM: Multiples of median
PRISMA-ScR: Preferred reporting items for systematic reviews and meta-analyses extension for scoping reviews
RBC: Red blood cells
Rh: Rhesus [factor]
