# Supplementary material for: Hemolytic disease of the fetus and newborn and Rhesus alloimmunization in Latin American countries: a scoping review
Source: BMC Pregnancy Childbirth. 2024 Dec 20;24:830. doi: 10.1186/s12884-024-07044-3 (PMC11660609; doi:10.1186/s12884-024-07044-3)
Supplement: Supplementary file 1 — Supplementary Material 1: Search strategies used in the databases. [file 12884_2024_7044_MOESM1_ESM.pdf]

Additional File 1: Search strategies

Search conducted on April 14, 2023, in the keyword field

No.	Search strategy	Ovid MEDLINE	OVID EMBASE
1	Fetal erythroblastosis OR Hemolytic Disease of the Newborn OR HDFN OR maternal-fetal alloimmunization.mp. OR RhD alloimmunization.mp. OR newborn hemolytic disease/ OR newborn hemolytic disease.mp	1370	2767
2	latin america.mp. OR exp Latin America/ OR americas.mp. OR Latin America countries.mp. OR Argentina.mp. OR Brazil.mp. OR Colombia.mp. OR Mexico.mp. OR Peru.mp.	229453	385630
3	1 AND 2	12	41
4	exp Epidemiology/ or epidemiology.mp. or exp incidence/ or prevalence.mp. or exp prevalence/ or morbidity.mp. or exp morbidity/ or mortality.mp. or exp mortality/ or burden of disease.mp.	2980276	5186198
5	socioeconomics'/de or 'cost benefit analysis'/de or 'cost of illness'/de or 'cost control'/de or 'economic aspect'/de or 'health care cost'/de or 'health care financing'/de or 'health economics'/de or 'hospital cost'/de or fiscal:ti.mp. or financial:ti.mp. or finance:ti.mp. or funding:ti.mp. or 'cost minimization analysis'.mp.	552	4166
6	quality adjusted life year.mp. or exp Quality-Adjusted Life Years/ or quality of life.mp. or exp "Quality of Life"/ or wellbeing.mp.	379704	843402
7	case study.mp. or case study/	1381846	189147
8	4 OR 5 OR 6	4492386	5905522
**9**	3 AND 7	7	20

Search strategy	LILACS	EPISTEMONIKOS	Value in Health
Fetal erythroblastosis OR Hemolytic Disease of the Newborn OR HDFN OR maternal-fetal alloimmunization OR RhD alloimmunization	13	129886	0
Argentina OR Brazil OR Colombia OR Mexico OR Peru	186353	39360	--
1 AND 2	13	0	0
